# Microgravity induces autophagy via mitochondrial dysfunction in human Hodgkin’s lymphoma cells

**DOI:** 10.1038/s41598-018-32965-3

**Published:** 2018-10-02

**Authors:** Ae Jin Jeong, Yoon Jae Kim, Min Hyuk Lim, Haeri Lee, Kumhee Noh, Byung-Hak Kim, Jin Woong Chung, Chung-Hyun Cho, Sungwan Kim, Sang-Kyu Ye

**Affiliations:** 1 Department of Pharmacology and Biomedical Sciences, Seoul National University College of Medicine, Seoul, 03080 Republic of Korea; 2 Biomedical Science Project (BK21PLUS), Seoul National University College of Medicine, Seoul, 03080 Republic of Korea; 3 Department of Biomedical Engineering, Seoul National University College of Medicine, Seoul, 03080 Republic of Korea; 4 Institute of Medical and Biological Engineering, Medical Research Center, Seoul National University College of Medicine, Seoul, 03080 Republic of Korea; 5 Ischemic/Hypoxic Disease Institute, and Seoul National University College of Medicine, Seoul, 03080 Republic of Korea; 6 Neuro-Immune Information Storage Network Research Center, Seoul National University College of Medicine, Seoul, 03080 Republic of Korea; 7 Interdisciplinary Program for Bioengineering, Graduate School, Seoul National University, Seoul, 08826 Korea; 8 Department of Biological Science, Dong-A University, Busan, 49315 Republic of Korea

## Abstract

Gravitational forces can impose physical stresses on the human body as it functions to maintain homeostasis. It has been reported that astronauts exposed to microgravity experience altered biological functions and many subsequent studies on the effects of microgravity have therefore been conducted. However, the anticancer mechanisms of simulated microgravity remain unclear. We previously showed that the proliferation of human Hodgkin’s lymphoma (HL) cells was inhibited when these cells were cultured in time-averaged simulated microgravity (taSMG). In the present study, we investigated whether taSMG produced an anticancer effect. Exposure of human HL cells to taSMG for 2 days increased their reactive oxygen species (ROS) production and NADPH oxidase family gene expression, while mitochondrial mass, ATPase, ATP synthase, and intracellular ATP levels were decreased. Furthermore, human HL cells exposed to taSMG underwent autophagy via AMPK/Akt/mTOR and MAPK pathway modulation; such autophagy was inhibited by the ROS scavenger *N*-acetylcysteine (NAC). These results suggest an innovative therapeutic approach to HL that is markedly different from conventional chemotherapy and radiotherapy.

## Introduction

The advent of human space exploration has produced novel research in to the effects of space travel on human health and diseases. In space, microgravity and cosmic radiation are considered the most consequential environmental factors^1,2^. Several studies have found that astronauts and experimental animals in space experience physiological changes owing to microgravity during and after space flights; the effects of microgravity include muscle atrophy, bone loss, immune dysregulation, and abnormal cellular functions^3–8^. Microgravity also affects major cellular functions such as cell growth, cell cycle, self-renewal and differentiation^7,9–12^. As such, it was hypothesized that microgravity has anticancer potential through cell growth inhibition, and many studies were conducted to investigate this notion^2,13–15^. Additionally, microgravity has been reported to cause cellular oxidative stress that leads to the production of reactive oxygen species (ROS), as well as endoplasmic reticulum stress^12,16–18^. However the mechanism by which microgravity elicits these cellular responses remain poorly understood.

Autophagy is a catabolic process that helps maintain cellular homeostasis through the degradation of bulk cytoplasm, long-lived proteins, and organelles in response to stresses such as nutrient deprivation, viral infection, and genotoxicity^19–21^. Recent evidence suggests that autophagy is an important mediator of pathological response and of the cell’s response to oxidative stress caused by ROS and reactive nitrogen species^22–25^. Autophagy involves the formation of double-membrane-bound structures called autophagosomes as initiated by the phosphoinositide 3-kinase (PI3K) type III-Atg6/Beclin-1 cascade^26,27^. The classic PI3K/Akt/mammalian target of rapamycin (mTOR) signaling pathway is an important negative regulator of autophagosome formation. Recent studies have found that activation of adenosine monophosphate-activated protein kinase (AMPK) results in autophagy via the negative regulation of mTOR and direct phosphorylation of Unc-51 like autophagy activating kinase 1 (ULK1)^28–30^. The role of autophagy induction in cells is depend on the nature of the stimulus as well as the cell type. In cancer therapy, autophagy increases cell migration, invasion, and chemoresistance, paradoxically autophagy can also induce cell death in response to certain stimuli and causes dysregulated cell energy metabolism^31–36^. Therefore, it is necessary to fully understand the function and mechanism of autophagy in cancer therapy.

Hodgkin’s lymphoma (HL) is a malignant tumor originating from B cells, while its precise cause is unknown, approximately 9,000 new patients are diagnosed annually in the United States^37^. HL is more common in males than in females, and most commonly occurs in individuals aged 15–40 or over 50 years but rarely in those under 10 years. If diagnosed found at an early stage, it can be treated with chemotherapy or radiotherapy, whereupon the 5-year survival rate is as high as 86%. Classical therapies have greatly improved the chance of cure, although their side effects are often severe^38–40^. Furthermore, recurrence after successful treatment is common^41–45^. Therefore, novel anticancer therapies that avoid the severe side effects and recurrence rates of existing modalities are needed.

In this study, we simulated a time-averaged microgravity environment to investigate if such a milieu produces an anticancer effect against human HL cells. The time-averaged simulated microgravity (taSMG) environment was produced using a clinostat as validated in a previous study^46^. The clinostat rotates in a manner that produces a constantly varying gravity vector in a non-repeating pattern, thereby producing a vector-free gravity environment by continuously averaging the vector. Using a clinostat can provide gravitational stress and microgravity-like effects in cells. In our previous studies, we confirmed that the proliferation of human HL cells is inhibited in a taSMG environment^46^. In this study, we hypothesized that taSMG produces cellular stress in human HL cells. We found that taSMG induces autophagy through mitochondrial dysregulation via the AMPK/Akt/mTOR and MAPK pathways.

## Results

### taSMG induces mitochondrial dysfunction in human HL cells

**Figure 1:**
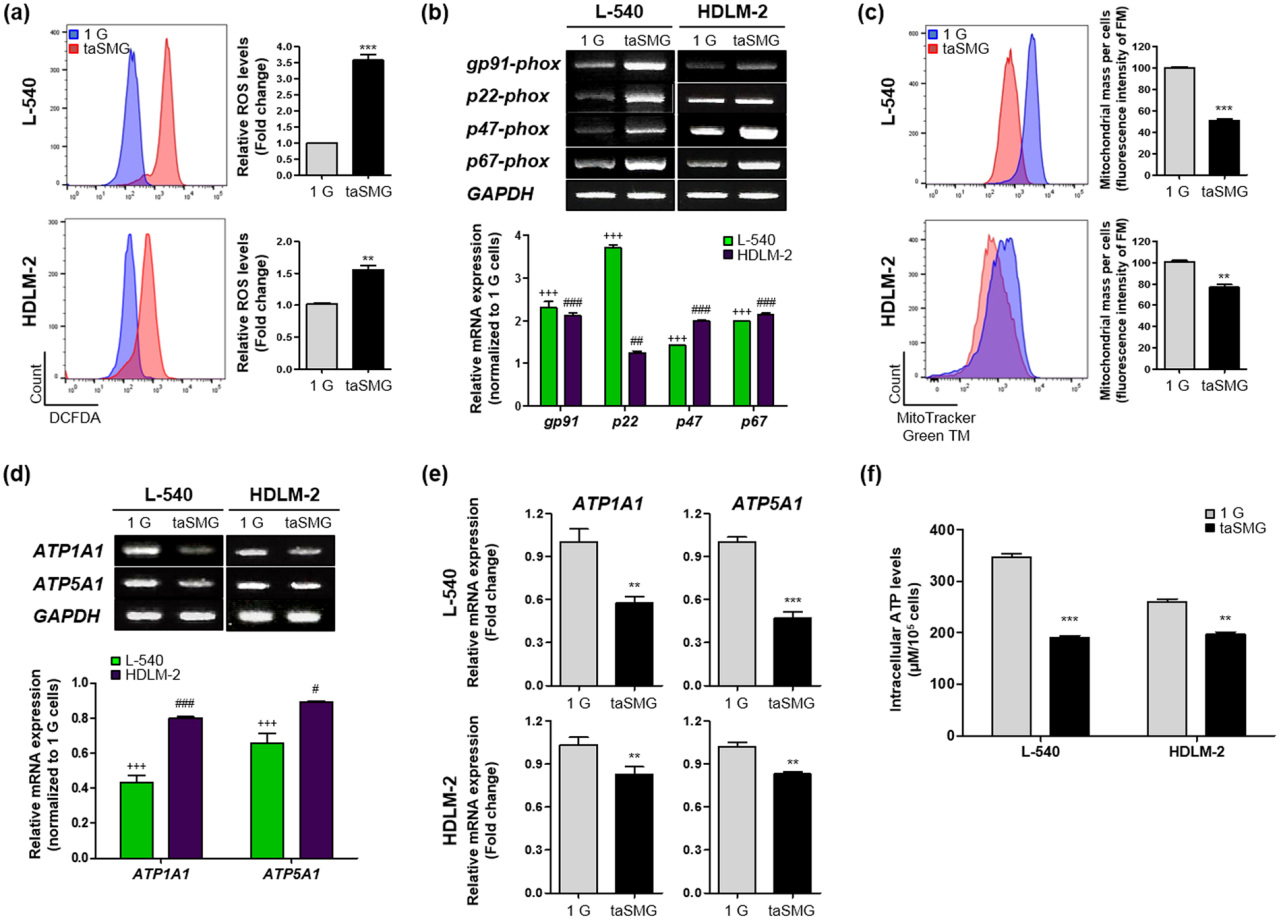
Time-averaged simulated microgravity induces mitochondrial dysfunction. Human HL cell lines (L-540 and HDLM-2) incubated in 1 G or taSMG conditions for 2 days were harvested. (**a**) ROS generation was determined by 2′,7′–dichlorofluorescin diacetate (DCFDA) staining. (**b**) RT-PCR analysis of NADPH oxidase family genes (*gp91-, p22, p47, and p67-phox*). (**c**) Mitochondrial mass was analyzed by MitoTracker^TM^ Green FM. (**d**) RT-PCR and (**e**) qRT-PCR analysis of ATPase (ATP1A1) and ATP synthase (ATP5A1) mRNA levels. (**f**) Analysis of intracellular ATP levels in L-540 and HDLM-2 cells. Data represent the mean ± SEM, n = 4. **p < 0.01 and ***p < 0.001 vs. the 1 G group. ^+++^*p* < 0.0001 vs. the 1 G group of L-540 cells. ^#^*p* < 0.05, ^##^*p* < 0.01 and ^###^*p* < 0.001 vs. the 1 G group of HDLM-2 cells. The grouping of gels cropped from different gels. 1 G: normal gravity conditions; taSMG: time-averaged simulated microgravity.

Microgravity has been reported to affect cell proliferation^47,48^. Our previous studies also showed that proliferation of human HL cells (L-540 and HDLM-2) is inhibited under taSMG conditions^46^. Since cell proliferation is mediated by mitochondrial regulation^49–51^, we investigated whether taSMG induces mitochondrial stress in human HL cells. We found that intracellular ROS levels were increased under taSMG compared to normal gravity, 1 G (Fig. 1a). As ROS is generated by nicotinamide adenine dinucleotide phosphate (NADPH) oxidase^52–54^, we also found that the expression levels of NADPH oxidase family genes (*gp91-, p22-, p47-, and p67-phox*) are higher under taSMG than under 1 G conditions (Fig. 1b). To observe changes in mitochondrial biogenesis, we measured the mitochondrial mass using MitoTracker labeling and found it to be significantly decreased under taSMG (Fig. 1c). Furthermore, the mRNA expression levels of ATPase (*ATP1A1*) and ATP synthase (*ATP5A1*) were notably lower than 1 G levels, as determined by RT-PCR and qRT-PCR (Fig. 1d,e). These findings confirmed that intracellular ATP levels were significantly lower under taSMG than 1 G (Fig. 1f). These results suggest that taSMG causes ROS generation and mitochondrial dysfunction in human HL cells.

### Mitochondrial dysregulation under taSMG leads to human HL cell autophagy

**Figure 2:**
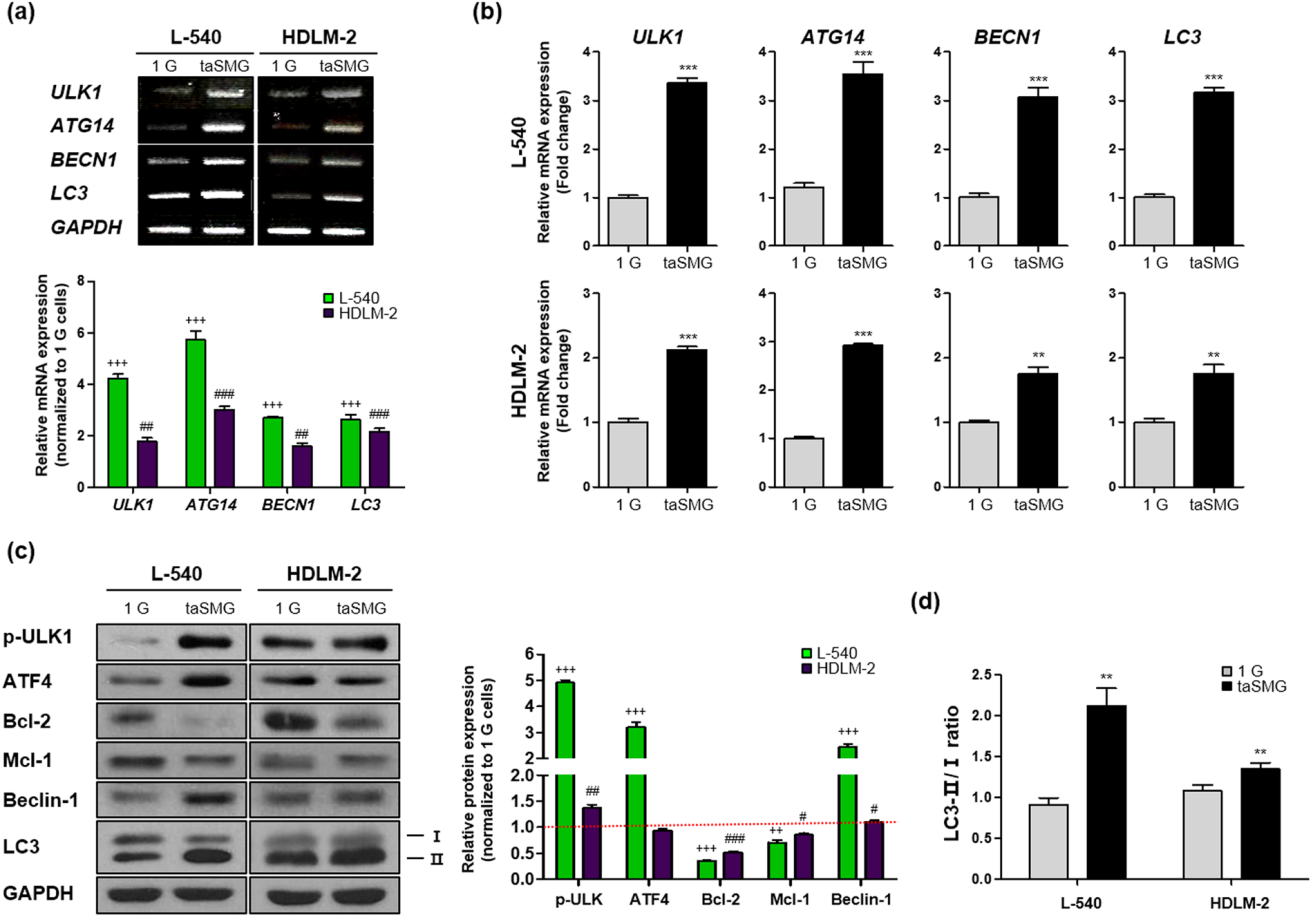
Time-averaged simulated microgravity contributes to induce autophagy. (**a**) RT-PCR and (**b**) qRT-PCR analysis of mRNA levels of autophagy-related genes. (**c**) Western blot analysis of autophagy-related proteins. GAPDH was used as the loading control. Densitometric analysis of protein expression was determined after normalization to GAPDH. The red dotted line indicates the base line. (**d**) Densitometric analysis of the LC3-II/I ratio was determined after normalization to GAPDH. Data represent the mean ± SEM, n = 4. ^+++^*p* < 0.0001 vs. the 1 G group of L-540 cells. ^#^*p* < 0.05, ^##^*p* < 0.01 and ^###^*p* < 0.001 vs. the 1 G group of HDLM-2 cells. **p < 0.01 and ***p < 0.001 vs. the 1 G group. The grouping of gels and blots cropped from different gels. 1 G: normal gravity conditions; taSMG: time-averaged simulated microgravity.

Mitochondrial dysfunction, as evidenced by increased ROS generation and reduced ATP levels, triggers autophagy^20,22,55^. Therefore, we measured the expression levels of the autophagy-related genes (*ULK1*, *ATG14*, *BECN1* and *LC3*); all were found to be upregulated under taSMG (Fig. 2a,b). Levels of phosphorylated ULK1, ATF4, Beclin-1, and microtubule-associated protein 1 light chain 3 (LC3) were increased, while expression of the Bcl-2 family proteins Bcl-2 and Mcl-1, which inhibit autophagy by directly binding to the BH3 domain of Beclin-1/Atg6, were decreased under taSMG conditions (Fig. 2c). Additionally, we found that the LC3-II/I ratio^56–58^, an indicator of autophagy, increased under taSMG (Fig. 2d). These results showed that taSMG contributes to the induction of autophagy in human HL cells.

### AMPK/Akt/mTOR and MAPK signaling are differentially regulated under taSMG

**Figure 3:**
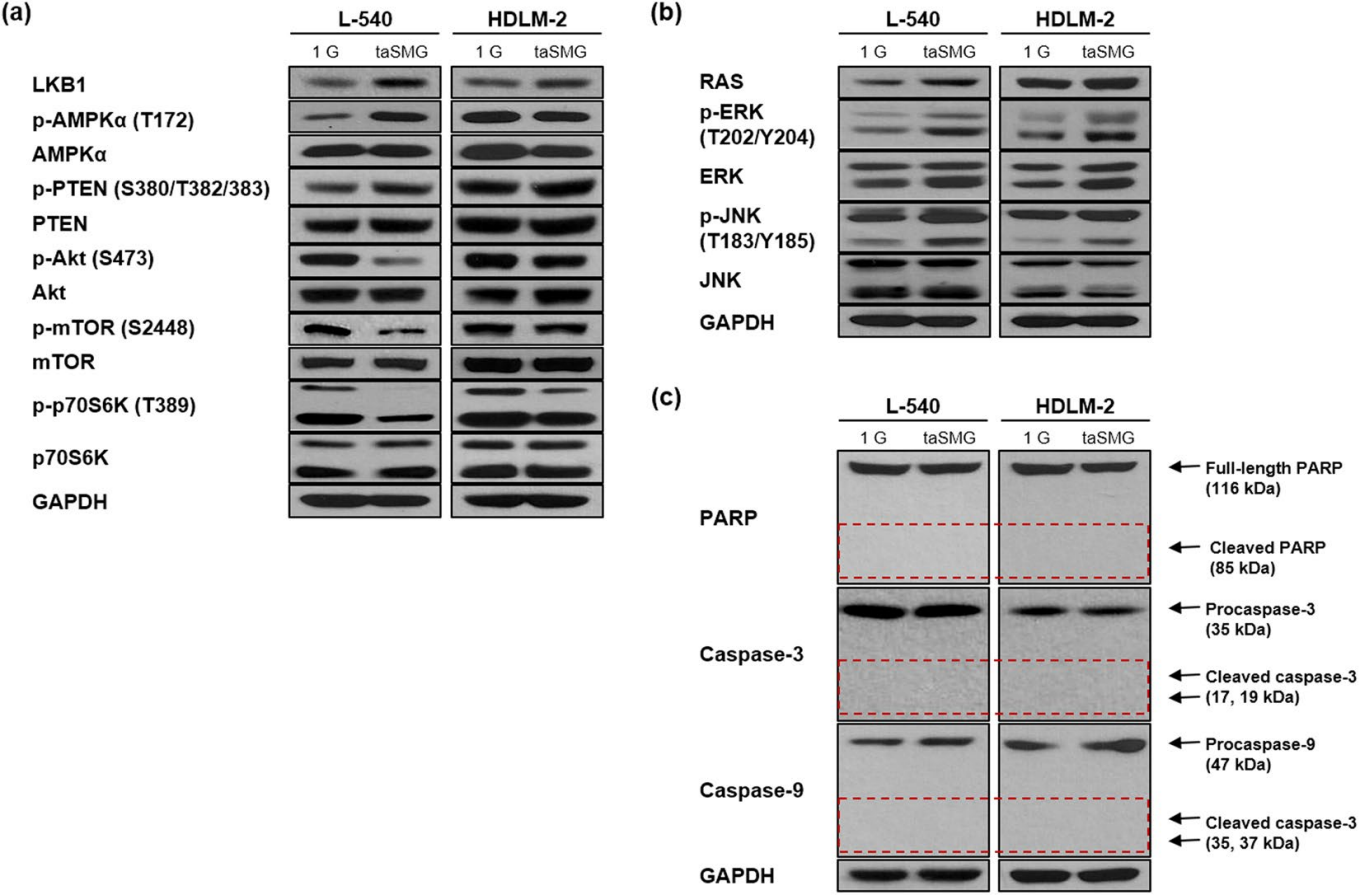
The regulation of AMPK/Akt/mTOR and MAPK pathways under time-averaged simulated microgravity. (**a**) Representative blots showing the phosphorylation levels of AMPK, PTEN, Akt, mTOR and S6K and total levels of LKB1, AMPK, PTEN, Akt, mTOR, and S6K in L-540 and HDLM-2 cells were determined by Western blotting. (**b**) Representative blots showing the activation levels of the MAPK pathway components RAS, ERK and JNK. (**c**) Representative blots showing apoptosis markers (PARP, caspase-3 and caspase-9). The dashed line is cleaved size that indicates apoptosis activation. All experiments were performed at least in quadruplicate. The grouping of gels cropped from different blots. 1 G: normal gravity conditions; taSMG: time-averaged simulated microgravity.

To further understand the mechanism of taSMG-induced autophagy, we investigated various cell signaling pathways by Western blotting. Since the liver kinase B1 (LKB1)/AMPK pathway is known to be activated under conditions of low intracellular ATP to inhibit cell growth and induce autophagy^59^, we have confirmed whether activation of these pathway under taSMG. We found that LKB1 and activated AMPK increased under taSMG (Fig. 3a). We also confirmed that activated AMPK inhibits the Akt/mTOR/S6K pathway (a negative regulator of autophagy) and activates the Akt suppressor protein PTEN (Fig. 3a). Previous studies have shown that ROS activates MAPK signaling, and that this elicits a variety of downstream signaling events^60–62^. To that end, we found that taSMG activates RAS, ERK, and JNK (Fig. 3b). Additionally, ROS-induced JNK and ERK activation are known to induce both autophagy and apoptosis^63^; however, no taSMG-induced apoptosis was observed (Fig. 3c). These results suggest that only autophagy is induced by modulating the AMPK/Akt/mTOR and MAPK signaling pathways under taSMG.

### ROS scavenging attenuates the mitochondrial dysfunction induced by taSMG

**Figure 4:**
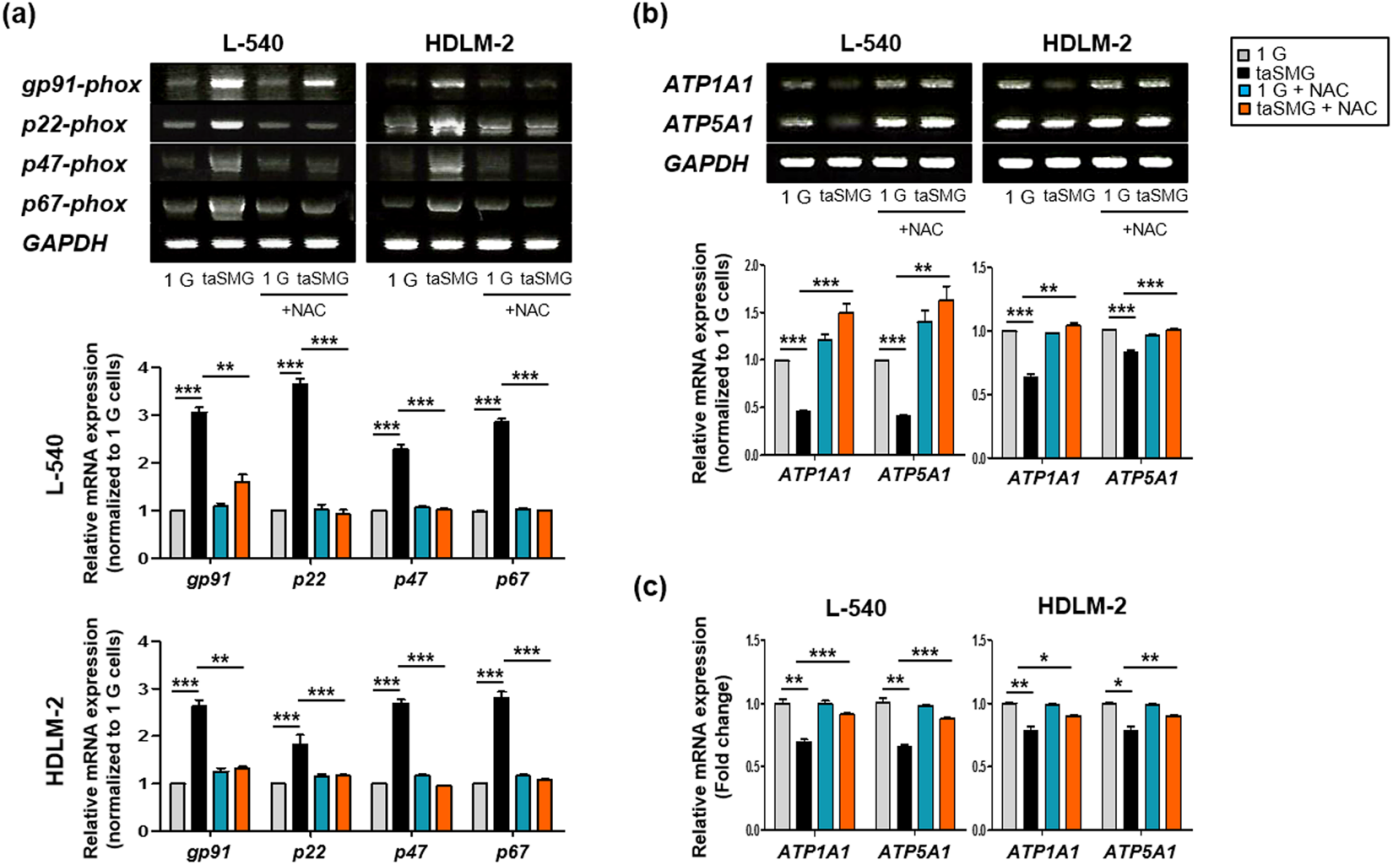
N-acetylcysteine protects mitochondrial dysfunction by time-averaged simulated microgravity. (**a**) RT-PCR data shows the mRNA levels of NADPH oxidase family genes. (**b**) RT-PCR and (**c**) qRT-PCR analysis of mRNA levels of ATPase and ATP synthase. Data represent the mean ± SEM, n = 4. *p < 0.05, **p < 0.01 and ***p < 0.001 vs. the 1 G group or taSMG. The grouping of gels cropped from different blots. 1 G: normal gravity conditions; taSMG: time-averaged simulated microgravity. Treatment of NAC proceeded under the same conditions.

We used the ROS scavenger *N-acetylcysteine* (NAC) to determine whether the abovementioned phenomena under taSMG can be inhibited. We treated the cells with NAC during clinostat operation and observed no increase in the mRNA levels of NADPH oxidase family genes (Fig. 4a). Using RT-PCR (Fig. 4b) and qRT-PCR (Fig. 4c), we also found that mRNA levels of ATPase and ATP synthase were not reduced under taSMG in the presence of NAC.

**Figure 5:**
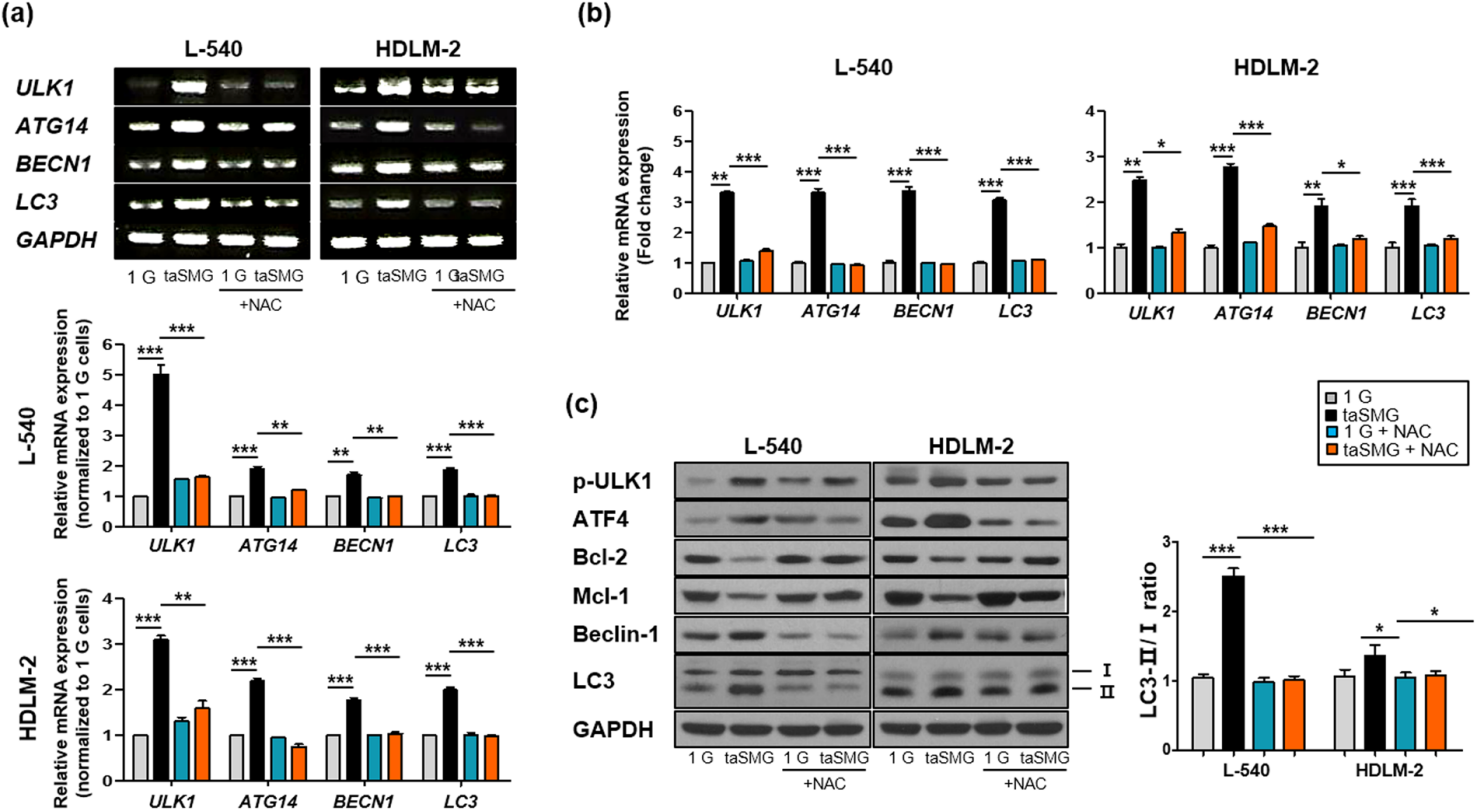
N-acetylcysteine prevents autophagy by time-averaged simulated microgravity. (**a**) RT-PCR and (**b**) qRT-PCR shows mRNA levels of autophagy-related genes. (**c**) Western blot analysis of protein levels of autophagy-related genes. (**d**) Densitometric analysis of LC3-II/I ratio was determined after normalization to GAPDH. Data represent the mean ± SEM, n = 4. *p < 0.05, **p < 0.01 and ***p < 0.001 vs. the 1 G group or taSMG. The grouping of gels cropped from different blots. 1 G: normal gravity conditions; taSMG: time-averaged simulated microgravity. Treatment of NAC proceeded under the same conditions.

In addition, we confirmed that taSMG-induced autophagy was inhibited by NAC treatment. In the presence of NAC, we found that there was no changes in autophagy-related mRNA (Fig. 5a,b) and protein levels, including the LC3-II/I ratio (Fig. 5c).

**Figure 6:**
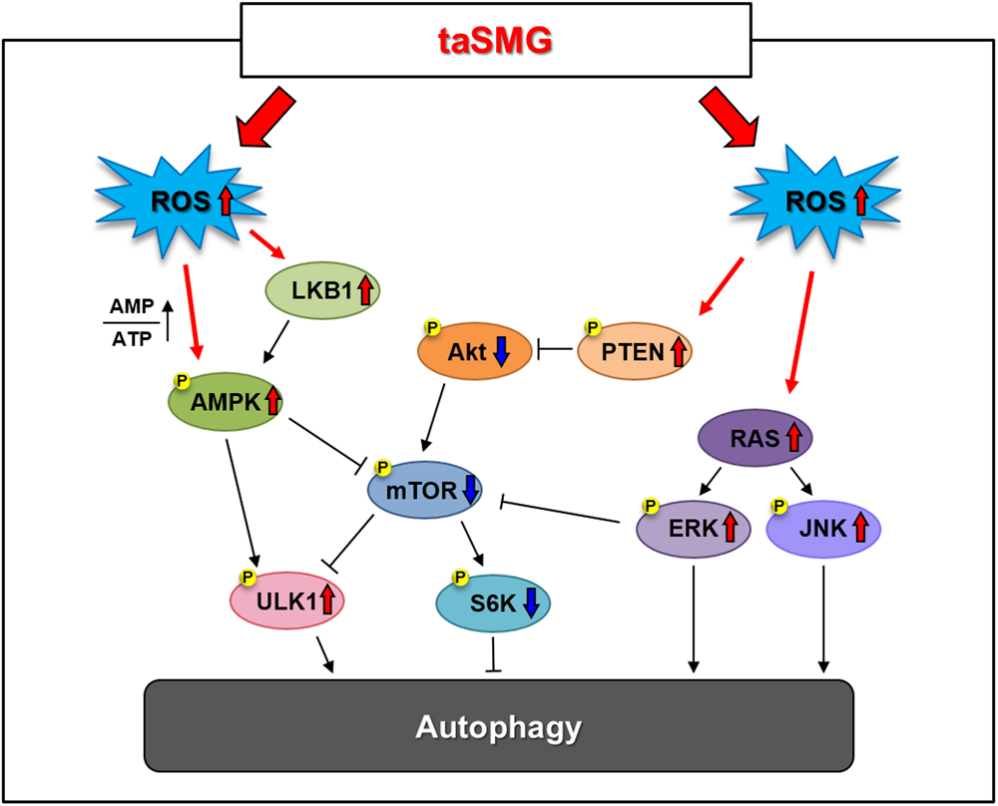
Schematic representation of the time-averaged simulated microgravity (taSMG)-induced autophagy mechanism in human Hodgkin’s lymphoma cells. taSMG induces the activation of AMPK, PTEN, and MAPK, and as well as the suppression of Akt, leading to the inhibition of mTOR activity. The downstream regulator S6K, which inhibits autophagy, is consequently suppressed, resulting in autophagy in human HL cells.

Taken together, these results indicate that autophagy was induced by mitochondrial dysfunction due to ROS generation under taSMG, suggesting that inhibition of ROS generation would prevent these phenomena (Fig. 6).

## Discussion

Primary chemoradiotherapy results in cure rates of 90% and 80% in HL patients with early- and advanced-stage disease, respectively^64^. However, these treatments lead to a significant risk of short- and long-term toxicities, which can also cause secondary malignancies^65,66^. Therefore, new therapies based on suppressing disease progression mechanisms, including molecule-specific inhibition are needed for successful treatment. We sought to investigate the use of microgravity as a potential new treatment for HL, given our previous findings that it inhibits cell proliferation^46^.

As taSMG can be achieved artificially, studies using this technique have advanced rapidly. Based on symptoms experienced by astronauts in the space such as muscle atrophy, bone loss, and immunodeficiency, microgravity has been investigated in the treatment of various diseases^2,4,6,7,10,17^. Our research team designed a clinostat that uses a specific algorithm aimed at randomizing the gravitational vector pattern and achieving a nullified time-averaged vector^46^.

Microgravity in space has been reported to cause oxidative stress such as ROS production^67–69^. However, the study of microgravity induced oxidative stress is not yet fully understood. In this study, we validated our hypothesis that taSMG induces oxidative cellular stress in human HL cells, as demonstrated by elevated ROS and impaired mitochondrial function.

NADPH oxidase-dependent ROS production is implicated in many physiologic and pathophysiologic processes. NADPH oxidase mediated ROS can alter parameters of signal transduction, mitochondrial damage, cell proliferation, cell death and autophagy^22,23,54,69^. We also found that taSMG upregulates NADPH oxidase family genes while decreasing the mitochondrial mass, and lowering ATPase, and ATP synthase levels, resulting in reduced intracellular ATP levels.

Autophagy mediates the bulk degradation of intracellular through lysosomal-dependent mechanisms and is necessary for the maintenance of cellular homeostasis^19,20,22^. Autophagy is also induced in response to oxidative stress caused by ROS and RNS^22,24,25^. We hypothesized that autophagy would occur due to increased ROS, and interestingly, autophagy was induced under taSMG.

Recently, some research reported that autophagy is induced in simulated microgravity and inhibits cancer proliferation and metastasis^18,21,70^. However, its molecular mechanisms are unclear yet. As an intracellular energy sensor, AMPK signaling serves as a mitochondrial function regulator to maintain energy homeostasis^29^. And recent studies have found that activation of AMPK results in autophagy through negative regulation of mTOR and phosphorylation of ULK1^27–30^. Interestingly, we showed that the mitochondrial dysfunction produced by taSMG promotes autophagy via modulation of the AMPK/Akt/mTOR and MAPK signaling pathways. Overall, the effect of taSMG was more prominent in L-540 cells than in HDLM-2 cells; L-540 cells have a higher proliferation rate, they are likely more sensitive to taSMG than HDLM-2 cells.

In fact, the role of autophagy in cancer remains controversial given that it varies according to the type of cancer. Autophagy may contribute to cancer progression by increasing cell migration and invasion; conversely, it can have an anticancer effect by decreasing cell proliferation, and promoting cancer cell death^28,31,34–36^.

Taken together, our research findings suggest that taSMG-induced autophagy following the induction of oxidative stress in human HL cells has an anticancer effect. For the scavenger of ROS generation, we confirmed that phenomena of oxidative stress and autophagy were prevented under taSMG.

We can’t know whether the effect of taSMG occurs in normal cells. Previous studies confirmed that proliferation of human dermal fibroblast (HDF) cells did not change under taSMG^46^. However, as we know that it is necessary to compare the effects of taSMG on normal lymphocytes and HL cells, we will proceed further study.

Our data ought to provide important insight concerning the effect of taSMG on cancer cells, as well as our understanding of human HL cell mechanisms. These findings could lead the way to promote new treatment methods for cancer patients.

## Materials and Methods

### Control of 3D Clinostat

3D clinostat, which consists of two perpendicular rotating axes, were developed and validated in our previous research^41^. The 3D clinostat provides taSMG, which is time-averaged acceleration (including gravitational, centrifugal, and tangential acceleration) less than 10^−3^G after 24 h operation. Coriolis, radial, and frictional forces were not considered in the simulation because angular velocities of two axes were set to sufficiently small values (inner frame: 0.683 rpm, outer frame: 0.913 rpm). Operation of 3D clinostat with designed angular velocity was proven to avoid repeated pattern of gravitational vector. For more detailed information, previously published paper can be helpful.

### Cell culture

Human Hodgkin’s lymphoma (HL) cell lines L-540 and HDLM-2 were obtained from the German Collection of Microorganisms and Cell Cultures (DSMZ, Braunschweig, Germany)^71^. The cell lines were maintained in RPMI 1640 (Life Technologies, USA) supplemented with 10% fetal bovine serum (FBS, Life Technologies, USA) and 1% penicillin/streptomycin solution (Life Technologies, USA) at 37 °C in 5% CO_2_. For cellular ROS inhibition, the cells were added with *N*-acetylcysteine (NAC, Sigma Aldrich, USA) at 10 mM in complete medium for operating clinostat. Simulation of microgravity using clinostat were previously described^46^. All experiments were performed after human HL cells were cultured under 1 G or taSMG condition for 2 days.

### ROS detection assay

For detection of cellular ROS, we used DCFDA cellular ROS detection assay kit (Abcam, UK). The collected cells were stained with 20 μM DCFDA in 1X buffer for 30 min at 37 °C. Without the washing procedure, stained cells were immediately carried out by flow cytometry (FACS) using FACS LSRFortessa (BD Bioscience, USA) at Ex488 nm/Em535 nm beam. Each determination was based on the mean fluorescence intensity of 10,000 cells.

### Mitochondrial mass analysis

Mitochondrial mass per cell was measured by flow cytometry using MitoTracker Green FM (Thermo Fisher Scientific, USA). Cells were collected, resuspended in 0.5 ml of PBS, and stained with 40 nM MitoTracker green (MTG) for 15 min at 37 °C in the dark. Cells were then washed with PBS, resuspended in FACS buffer (eBioscience, USA), and added 200 ng/ml of DAPI (Sigma Aldrich, USA). Stained cells were analyzed using FACS LSRFortessa at Ex488 nm/Em535 nm beam. Each determination was based on the mean fluorescence intensity of 25,000 cells.

### Reverse transcription (RT)-PCR and quantitative real-time RT-PCR (qRT-PCR)

Total RNA were extracted using a RNAiso Plus reagent (Takara, Japan) and cDNA was synthesized using ReverTra Ace qPCR RT Master Mix kit (TOYOBO, Japan). Quantitative real time-PCR was performed using the SYBR Green PCR mix (Applied Biological Material, Canada) with an Applied Biosystems 7300 Real-time PCR system (Life Technologies, USA) and the raw data were analyzed using comparative Ct quantification. All primers were purchased from Qiagen (Qiagen, USA). The as basic PCR amplification conditions were 58 °C annealing temperature and 35 cycles.

### ATP assay

Levels of intracellular ATP was measured using an ATP Bioluminescence assay kit (Roche, Switzerland) according to manufacturer’s instruction. Cells were collected and heated tubes to at least 95 °C for 7 min. To remove pellet cell debris, spin down at 14000 RPM for 3 min. And then each sample were transferred to a black 96 well plate and quickly added luciferase reagent to each well. Luminescence was measured at 0.1 sec/well using the luminescence program.

### Western blot

Cells were lysed in a lysis buffer (50 mM Tris-HCl, pH 7.4, 350 mM NaCl, 0.5% NonidetP-40, 10% glycerol, 0.1% SDS, and 1% Triton X-100). The lysates were centrifuged at 13200 rpm for 10 min at 4 °C and protein amounts were quantified using a Bio-Rad protein assay (Bio-Rad, USA). Proteins were separated by SDS-poly acrylamide gel electrophoresis (SDS-PAGE) and transferred onto nitrocellulose membranes (Whatman, Atlanta, USA). The membranes were blocked in blocking buffer (5% skim milk in 150 mM NaCl, 25 mM Tris-HCl, pH 7.4, and 0.1% Tween 20) and subsequently incubated with specific primary antibodies for the target molecules. The membranes were then washed with Tris-buffered saline containing 0.1% Tween 20 (TBS-T) and further incubated with horseradish peroxidase (HRP)-conjugated secondary antibodies for 1 h at room temperature. After washing with TBS-T, the signals were visualized using the ECL Plus Western blotting substrate (Thermo Fisher Scientific, USA).

### Statistical analysis

All experiments were performed by more than four times. The results are represented as means with standard error of the mean (SEM). Statistical significance was determined based on a two-tailed Student’s t-test and analyzed using Graph Pad Prism 6 (Graph Pad Software, INC., USA).
